# *Mycoplasma pneumoniae* infections, 11 countries in Europe and Israel, 2011 to 2016

**DOI:** 10.2807/1560-7917.ES.2020.25.2.1900112

**Published:** 2020-01-16

**Authors:** Michael L Beeton, Xu-Sheng Zhang, Søren A Uldum, Cécile Bébéar, Roger Dumke, Karolina Gullsby, Margareta Ieven, Katherine Loens, Ran Nir-Paz, Sabine Pereyre, O Brad Spiller, Victoria J Chalker, the ESCMID Study Group for Mycoplasma and Chlamydia Infections (ESGMAC) Mycoplasma pneumoniae subgroup

**Affiliations:** 1 Department of Biomedical Sciences, Cardiff Metropolitan University, Cardiff, United Kingdom; 2 Public Health England, London, United Kingdom; 3 Department of Bacteria, Parasites and Fungi, Statens Serum Institut, Copenhagen, Denmark; 4 USC-EA 3671, Mycoplasmal and Chlamydia Infections in Humans, University of Bordeaux, Bordeaux, France; 5 TU Dresden, Dresden, Germany; 6 Centre for Research and Development, Uppsala University/Region Gävleborg, Gävle, Sweden; 7 Antwerp University Hospital Edegem, Belgium; 8 Department of Clinical Microbiology and Infectious Diseases, Hadassah-Hebrew University Medical Center, Jerusalem, Israel; 9 Department of Medical Microbiology, Division of Infection and Immunity, Cardiff University, School of Medicine, Cardiff, United Kingdom; 10 ESCMID Study Group for Mycoplasma and Chlamydia Infections (ESGMAC) *Mycoplasma pneumoniae* subgroup members are listed at the end of the article

**Keywords:** Mycoplasma pneumoniae, epidemiology, diagnostics, surveillance

## Abstract

**Background:**

*Mycoplasma pneumoniae* is a leading cause of community-acquired pneumonia, with large epidemics previously described to occur every 4 to 7 years.

**Aim:**

To better understand the diagnostic methods used to detect *M*. *pneumoniae*; to better understand *M. pneumoniae* testing and surveillance in use; to identify epidemics; to determine detection number per age group, age demographics for positive detections, concurrence of epidemics and annual peaks across geographical areas; and to determine the effect of geographical location on the timing of epidemics.

**Methods:**

A questionnaire was sent in May 2016 to *Mycoplasma* experts with national or regional responsibility within the ESCMID Study Group for Mycoplasma and Chlamydia Infections in 17 countries across Europe and Israel, retrospectively requesting details on *M. pneumoniae-*positive samples from January 2011 to April 2016. The Moving Epidemic Method was used to determine epidemic periods and effect of country latitude across the countries for the five periods under investigation.

**Results:**

Representatives from 12 countries provided data on *M. pneumoniae* infections, accounting for 95,666 positive samples. Two laboratories initiated routine macrolide resistance testing since 2013. Between 2011 and 2016, three epidemics were identified: 2011/12, 2014/15 and 2015/16. The distribution of patient ages for *M. pneumoniae-*positive samples showed three patterns. During epidemic years, an association between country latitude and calendar week when epidemic periods began was noted.

**Conclusions:**

An association between epidemics and latitude was observed. Differences were noted in the age distribution of positive cases and detection methods used and practice. A lack of macrolide resistance monitoring was noted.

## Introduction

*Mycoplasma pneumoniae* is a major cause of respiratory infection in humans and macrolide antibiotics, such as azithromycin, are used as the first-line of treatment in many countries. The bacterium is transmitted from person-to-person by respiratory droplets with the incubation period ranging from 4 days to 3 weeks [1]. Because of *M. pneumoniae*’s intrinsic resistance to many antibiotics, including all cell wall inhibitors, macrolide antibiotics such as azithromycin and clarithromycin are the drug of choice for treatment. In cases of suspected infection in immunocompromised individuals, bactericidal fluoroquinolones may be considered. Tetracyclines are an alternative for treatment of adults with possible macrolide-resistant *M. pneumoniae* infections. Prudent use of antibiotics has been urged for all cases of *M. pneumoniae* infection because of worldwide reports of macrolide resistance, which have been reported as ranging from 0.2% in Sweden to more than 90% in China [2-5].

*M. pneumoniae* infections show seasonal variation. In temperate climates, the number of infections peak during the latter months of the years, with epidemic periods every 4 to 7 years on average [6-8]. The most recent survey in 2012 by Lenglet et al indicated that some countries in the European Union and European Economic Area experienced an increase in *M. pneumoniae* cases in 2011 whereas others did not, indicating that a universal geographic increase had not occurred [5]. Little is understood about the transmission of *M. pneumoniae* within populations and several factors have been postulated to account for transmission dynamics, including the immunity level of the population, the bacterial population based on the P1 adhesin type, the age and extent of mixing of children in educational settings.

Methodologies for detection of *M. pneumoniae* include nucleic acid amplification tests (NAAT), serology and culture with varying sensitivities and specificities. There is no international standard material for quality control detection in assays, although external quality control schema exist for some methodologies (NAAT). There are no internationally defined guidelines on the requirements for surveillance of *M. pneumoniae*, macrolide resistance testing and surveillance, reference system structure, routine testing and bacterial strain discrimination. However, a few countries such as France and the United States (US) have surveillance within specific regions and national surveillance is seen in countries such as Denmark [9] and Japan, the latter of which has maintained an active surveillance system for this pathogen for some time [10]. Overall, laboratory confirmed cases and surveillance data regarding the number of cases and reported cases of macrolide resistance are likely to be underestimated. This is further confounded because an undefined proportion of patients will have mild disease or may be carriers within community settings, without active testing to confirm the infection. Further underestimation is likely to occur from patients receiving empirical treatment in the absence of laboratory-confirmed infection with *M. pneumoniae*.

In response to an increase in infection seen in several countries in 2016, the European Society of Clinical Microbiology and Infectious Diseases (ESCMID) Study Group for Mycoplasma Infections (ESGMI), now called the Study Group for Mycoplasma and Chlamydia Infections (ESGMAC), established this study [9,11]. The purpose of the study was to gain a greater understanding of the diagnostic methods used to detect *M. pneumoniae*; gain a greater understanding of the testing and surveillance in use for *M. pneumoniae* (macrolide resistance, seasonality); to identify epidemics; to determine detections per age group, age demographics for positive detections and concurrence of epidemics and annual peaks across geographical areas; and to determine the effect of geographical location on timing of epidemics.

## Methods

### Study type, data collection and analysis

ESGMI conducted a retrospective email-based survey in May 2016 of ESGMI members in countries in Europe and Israel, asking members to describe existing laboratory-confirmed case data for *M. pneumoniae* infection. This retrospective study involved sending an email-based survey to 18 experts collating laboratory-confirmed documented detections of *M. pneumoniae* from national laboratory and surveillance institutions or, if not available, other regional laboratory and surveillance institutions*.* Mycoplasma experts invited to participate in the study were either active members of ESGMI or authors listed in the previous study by Lenglet et al [5]. Participants were invited to join the study and provide the number of detections confirmed by nucleic acid amplification test (NAAT), serology, culture and total overall between weeks commencing 3 January 2011 to 24 April 2016. Positive results and, if available, negative results were also collated. For Germany and France, only regional data were available.

Additional information was requested, including what diagnostic methods were used to detect *M. pneumoniae*; whether surveillance for *M. pneumoniae* was in use; if macrolide resistance was being monitored by countries; and *M. pneumoniae* detection number per age group.

Data from each participating country was collated and aggregated to give total number of detections per age group and four weekly moving averages of detections per country and overall where possible. We did not request information on the sex of patient from which detections were made. Total weekly data were subcategorised by age group: 0 to 4 years, 5 to 9 years, 10 to 14 years, 15 to 24 years, 25 to 44 years, 45 to 64 years, ≥ 65 years or unknown.

### Case definition

Cases of *M. pneumoniae* infection were defined by local practice. Because of local variation, this study collated information on *M. pneumoniae* detections, not cases.

### De-duplication and exclusion criteria

Because of the heterogeneous nature of *M. pneumoniae* data collection from each country, defining study-wide de-duplication criteria was not feasible. Participants were therefore asked to detail if data with duplicate samples from the same patient (e.g. with NAAT and serology) was included as a single category and if possible, to include as serology. Specific exclusion criteria were also not set for similar reasons stated above. Responses for de-duplication and exclusion criteria are listed in Supplementary Table S1.

### Characteristics of epidemics using the Moving Epidemic Method

To determine the characteristic properties of *M. pneumoniae* epidemics across the 12 countries for which data were provided on *M. pneumoniae* positivity, the Moving Epidemic Method (MEM) was used [12]. An epidemic slope threshold of 2% was chosen and used to determine the pre-epidemic period, epidemic period and post-epidemic period for the five periods coinciding with annual peaks spanning week 19 of the first year through week 18 of the following year. These data were used to calculate the epidemic duration for each country, as well as the percentage of positive samples that were identified within this period. Data generated from the MEM model, such as week number in which epidemics began, were used to correlate the onset of epidemics with the geographical location of each country. Statistical analysis for generating p values and calculation of r^2^ were performed with GraphPad Prism 5.0.

### Ethics statement

This retrospective study involved collation of anonymised surveillance data for epidemiological analysis. Ethical approval was not required and no patient identifiers were included in the study.

## Results

Of the 18 countries approached, 11 countries across Europe and Israel, provided information regarding *M. pneumoniae*. For Cyprus, Malta, the Netherlands, Poland, Slovakia and Spain, *M. pneumoniae* national testing and surveillance systems were not in place or a response with data was not received within the timeframe of the study.

### *Mycoplasma pneumoniae* detection methods

During the study period, a total of 95,666 detections of *M. pneumoniae* were confirmed from participating countries (Table 1). The method of *M. pneumoniae* detection varied between the 12 countries. Two countries, Denmark and Israel, reported exclusively NAAT use; two countries, Greece and Ireland, reported serology exclusively; five countries, Germany, Hungary, Norway, Sweden and the United Kingdom (excluding Northern Ireland) used a combination of NAAT and serology; one country, Slovenia, used NAAT in combination with culture; and two countries, Belgium and France, used all three methods. No country relied on culture alone (Table 1).

**Table 1:** *Mycoplasma pneumoniae* detection methods, percent of positive samples and macrolide resistance monitoring by country, 11 countries in Europe and Israel, 2011–2016

Country	Nucleic acid amplification test		Culture		Serology		Total number of positive samples	Total number of negative samples	Percent of positive samples (%)	Macrolide resistance monitoring
	Performed	Number of positive detections	Performed	Number of positive detections	Performed	Number of positive detections				
Belgium	Yes	894	Yes	49	Yes	12,047	21,094^a^	ND	NA	Only monitored when samples test positive at the NRC. No testing in sentinel laboratories.
Denmark	Yes	20,081	No	NA	No	NA	20,081	264,770	7.0	No routine surveillance system in place. In 2010/11, 2011/12 and 2015/16, 809 samples were examined identifying 13 macrolide resistance-associated mutations (1.5%). Samples are investigated upon physician request.
France	Yes	92	Yes	53^b^	Yes	298	390	7,463	5.0	Performed on all clinical specimens detected as *M. pneumoniae*-positive by NAAT since 2013 [13].
Germany	Yes	127	No	NA	Yes	316	443	10,143	4.2	No comment
Greece	No	NA	No	NA	Yes	140	140	1,498	8.5	Information not provided
Hungary	Yes	17	No	NA	Yes	1,117	1,134	6,109	15.7	Information not provided
Ireland	No	NA	No	NA	Yes	535	535	2,853	15.8	Information not provided
Israel	Yes	848	No	NA	No	NA	848	5,309	13.8	No active monitoring.
Norway	Yes	13,980	No	NA	Yes	10,678	24,658	ND	NA	Information not provided
Slovenia	Yes	1,172	Yes	827	No	NA	1,172	8,872	11.7	Only upon physician request
Sweden	Yes	9,499	No	NA	Yes	10,024	19,523	169,501	10.3	No active monitoring.
United Kingdom (excluding Northern Ireland)	Yes	385	No	NA	Yes	5,263	5,648	ND	NA	No national system. All positive samples referred to PHE are tested.
**Total**	**NA**	**47,095**	**NA**	**876**	**NA**	**40,418**	**95,666**	**476,518**	**NA**	**NA**

NA: not applicable; NAAT: Nucleic acid amplification test; ND: no data available; NRC: National Reference Centre; PHE: Public Health England.

^a^ Method of detection not known for sentinel laboratories.

^b^ Not included in the overall total for the purpose of de-duplication as these 53 samples were already listed in NAAT or serological counts.

Responses were received to state *M. pneumoniae* testing and surveillance is not in place on a national scale or case data were not available within the timespan of the survey from European Society of Clinical Microbiology and Infectious Diseases (ESCMID) Study Group for Mycoplasma Infections (ESGMI) members from Cyprus, Malta, the Netherlands, Poland, Slovakia or Spain.

The greatest number of positive samples were reported using NAAT (47,095; 49%) followed by serology (40,418; 42%). Only 876 (1%) samples were reported positive by culture and 7,277 (8%) of tests were not specified. Norway contributed the greatest number of *M. pneumoniae-*positive detections to the total figure (24,658; 26%) and Greece the lowest (140; 0.1%). De-duplication data were determined at country level (Supplementary Table S1).

### Macrolide resistance monitoring

With regards to active monitoring of macrolide resistance, five countries did not comment; Belgium noted that monitoring was only carried out on positive samples identified at the National Reference Laboratory, but not at sentinel laboratories; Slovenia noted that macrolide resistance determination was carried out only upon physician request; in this study, Denmark stated that NAAT-positive samples from three recent *M. pneumoniae* epidemic periods were investigated, finding a low level (1.5%) of macrolide resistance, and that clinical samples are investigated on request from physicians; the Mycoplasmal and Chlamydia Infections in Humans Research Department, University of Bordeaux, France initiated national systematic monitoring of all NAAT-positive clinical specimens in 2013 using an in-house published method [13]. In 2017, England and Wales introduced monitoring of all positive NAAT samples referred to the National Reference Laboratory. Two countries stated that no monitoring for resistance was in place (Table 1).

### Total number of detections and seasonality

The distribution and seasonality of the 95,666 detections from the 12 countries across the study period was determined. To account for weekly bias in reporting, data were converted to four weekly moving average. The greatest number of positive samples from the four weekly moving average data was 1,759 positive detections during week 48 of 2011.

**Figure 1:**
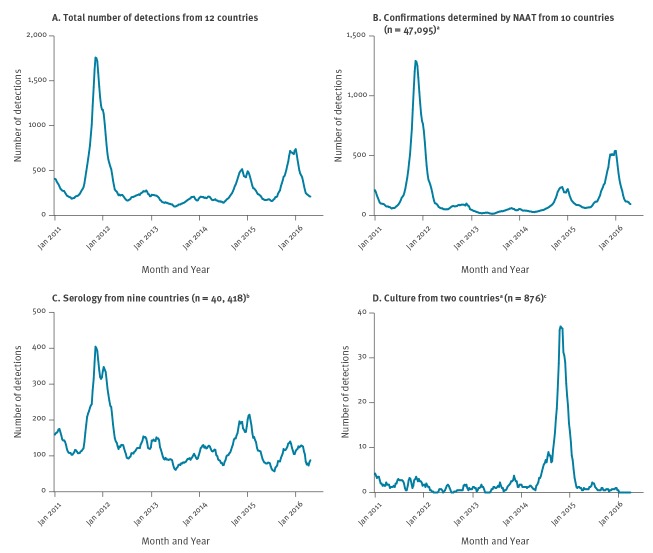
Four weekly moving average data on *Mycoplasma pneumoniae* infections by detection methods, 11 countries in Europe and Israel, 2011–2016

Detection of *M. pneumoniae* by NAAT correlated with the overall detections (Figure 1A and Figure 1B). Detection of *M. pneumoniae* by culture accounted for the lowest number of positive samples per methodology; 1% of the total positive samples. Detection using serology was the second most common method for detecting *M. pneumoniae-*positive patients (Figure 1C). The four weekly moving average for detection by culture (Figure 1D) was less than five positive samples for all reporting weeks with the exception of Slovenia in the 2015 season when a maximum average of 52 positive samples was identified.

NAAT: nucleic acid amplification tests.

^a^ Ten countries: Belgium, Denmark, France, Germany, Hungary, Israel, Norway, Slovenia, Sweden and the United Kingdom (excluding Northern Ireland).

^b^ Nine countries: Belgium, France, Germany, Greece, Hungary, Ireland, Norway and Sweden and the United Kingdom (excluding Northern Ireland).

^c^ Two countries: Belgium and Slovenia. Culture data from France not included as it was duplicated in NAAT and serological data.

### Epidemic periods based on the Moving Epidemic Method

**Figure 2:**
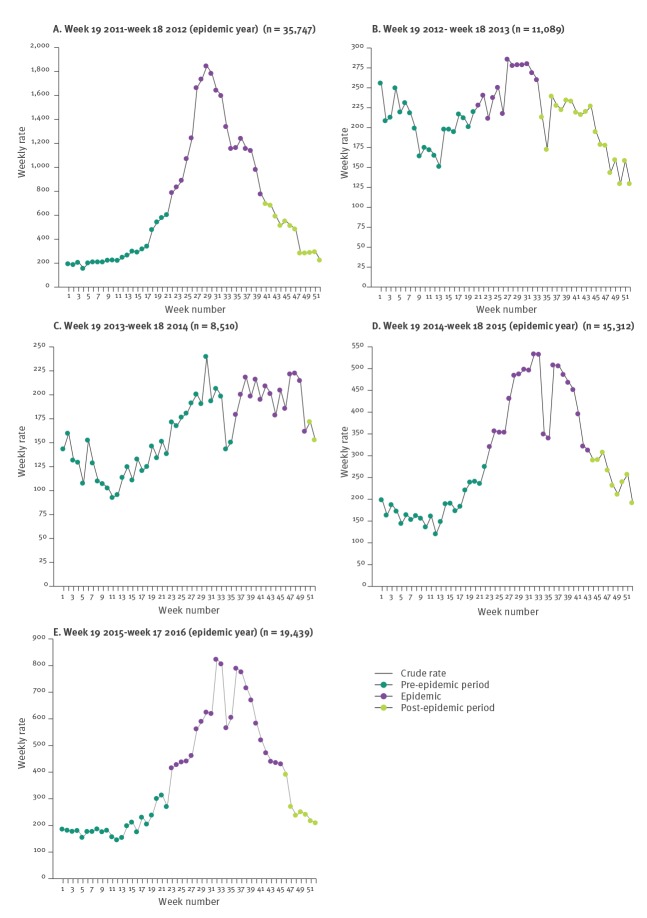
Analysis of *Mycoplasma pneumoniae* epidemic periods using the Moving Epidemic Method, 11 countries in Europe and Israel, 2011–2016

Analysis of detections during the annual periods was carried out using MEM (Figure 2). For the five annual periods described, we noted 35,747; 11,089; 8,510; 15,312; 19,439 detections for the periods 2011/12, 2012/13, 2013/14, 2014/15 and 2015/16, respectively. Three epidemics were detected between 2011/12, 2014/15 and 2015/16, in which 67%, 59% and 68% of each period’s detections were identified during the calculated epidemic period, respectively. Epidemics had longer duration, 19, 21 and 23 weeks, respectively, compared with the duration observed during annual seasonal peaks of infection (non-epidemic periods). In 2012/13, the duration was 13 weeks with 30% of total detections and in 2013/14, 15 weeks with 35% of total detections. For countries providing the total number of negative samples, the percentage of positive samples identified during the pre-epidemic, epidemic and post-epidemic period was calculated for the epidemic periods of 2011/12, 2014/15 and 2015/16 (Table 2). For all periods, there was a greater percentage of positive samples during the epidemic period than in the pre-epidemic period.

MEM: Moving Epidemic Method.

Week numbers represent epidemic week period (week 1 represents calendar week 19). Green dots represent pre-epidemic period, purple dots represent epidemic period and yellow dots represent post-epidemic period, as calculated by the MEM.

**Table 2:** Proportion of total samples positive for *Mycoplasma pneumoniae* during the pre-epidemic, epidemic and post-epidemic periods, 8 countries in Europe and Israel, epidemic periods 2011/12, 2014/15 and 2015/16^a^

Country	2011/12										2014/15										2015/16									
	Pre-epidemic		Epidemic		Post-epidemic		Total across all periods		Detection per peak week during epidemic period		Pre-epidemic		Epidemic		Post-epidemic		Total across all periods		Detection per peak week during epidemic period		Pre-epidemic		Epidemic		Post-epidemic		Total across all periods		Detection per peak week during epidemic period	
	n/N	%	n/N	%	n/N	%	n/N	%	n/N	%	n/N	%	n/N	%	n/N	%	n/N	%	n/N	%	n/N	%	n/N	%	n/N	%	n/N	%	n/N	%
Denmark	920/7,784	12	6,932/47,522	15	804/15760	5	8,656/71,066	12	185/879	21	291/12,171	2	1,206/23,252	5	204/7,876	3	1,701/43,299	4	90/1238	7	1,120/18,340	6	5,751/52,827	11	712/16,139	4	7,583/87,306	9	546/3, 457	16
France	71/862	8	41/410	10	27/583	5	139/1,855	7	7/37	19	32/786	4	17/208	8	19/381	5	68/1,375	5	6/44	14	28/690	4	8/86	9	22/506	4	58/1,282	5	4/29	14
Germany	38/833	5	33/360	9	37/669	6	108/1,862	6	7/44	16	8/447	2	77/1,373	6	4/248	2	89/2,068	4	7/39	18	22/917	2	13/293	4	19/1,639	1	54/2,849	2	4/46	9
Greece	NA	NA	18/110	16	16/224	7	34/334	10	5/8	63	NA	NA	9/27	33	28/304	9	37/331	11	3/6	50	0/15	0	2/11	18	6/179	3	8/205	2	2/3	67
Hungary	15/154	10	32/169	19	105/942	11	152/1,265	12	7/20	35	75/442	17	186/760	24	54/367	15	315/1,569	20	5/12	42	96/570	17	50/220	23	59/544	11	205/1,334	15	10/30	33
Ireland	53/290	18	77/261	30	15/78	19	145/629	23	3/6	50	28/263	11	31/153	20	17/144	12	76/560	14	4/10	40	66/374	18	39/153	25	17/73	23	122/600	20	8/12	67
Israel	84/572	15	81/346	23	NA	NA	165/918	18	19/55	35	57/499	11	87/597	15	NA	NA	144/1,096	13	5/17	29	106/1052	10	82/687	12	3/36	8	191/1,775	11	11/45	24
Slovenia	32/389	8	31/349	9	12/413	3	75/1,151	7	4/18	22	166/775	21	583/1,866	31	83/1,037	8	832/3,678	23	38/78	49	NA	NA	29/469	6	28/1,447	2	57/1,916	3	5/27	19
Sweden	1,389/7,389	19	5,356/28,226	19	859/10,034	9	7,604/45,649	17	186/686	27	863/10,734	8	1,447/17,242	8	501/8,729	6	2,811/36,705	8	100/950	11	500/7,955	6	1,356/12,414	11	967/14,066	7	2,823/34,435	8	98/731	13
**Total**	**2,602/18,273**	**14**	**12,601/77,753**	**16**	**1,875/28,703**	**7**	**17,078/124,729**	**14**	**423/1,753**	**24**	**1,520/26,117**	**6**	**3,643/45,478**	**8**	**910/19,086**	**5**	**6,073/90,681**	**7**	**258/2,394**	**11**	**1,938/29,913**	**6**	**7,330/67,160**	**13**	**1,833/34,629**	**5**	**11,101/13,1702**	**8**	**688/4,380**	**16**

NA: not applicable.

^a^ Only those countries reporting both positive and negative sample data are included.

### Distribution of *Mycoplasma pneumoniae* positivity by age

**Figure 3:**
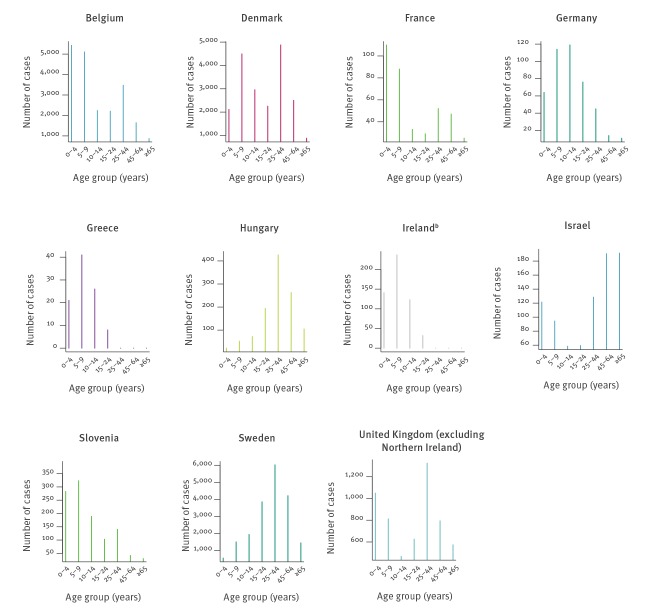
Number of *Mycoplasma pneumoniae* detections by age group, 10 countries in Europe^a^ and Israel, 2011–2016 (n = 70,191)

The distribution of *M. pneumoniae* positivity by age group was determined for 11 countries (Figure 3) as data obtained from Norway was not subcategorised by age. Detections per age group varied, and infections were noted in all age groups (0–4 years of age 9,832; 5–9 years of age 12,957; 10–14 years of age 8,144; 15–24 years of age 9,311; 25–44 years of age 16,281; 45–64 years of age 9,586; ≥ 65 years of age 4,080; total with specified age group 70,191). Even distribution across all age groups was not noted in the data from any country. Country-specific age data revealed three distinct patterns. Four countries, Germany, Greece, Ireland and Slovenia, showed skewing of positive samples to younger patients (< 10 years of age), whereas two countries, Hungary and Sweden, showed skewing towards older patients (> 25 years of age). Five countries reported a bimodal distribution: Belgium, Denmark, France, Israel and the United Kingdom (excluding Northern Ireland), reported a bimodal distribution. Of note, data for Ireland was not available for cases 25 years of age and above.

**^a^** Age-specific data were not available for Norway.

^b^ Ireland did not test for *M. pneumoniae* in patients 25 years of age and above.

### Correlation between latitude and epidemic period onset

**Figure 4:**
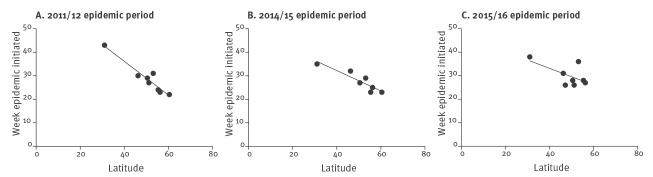
Correlation between country latitude and epidemic week for *Mycoplasma pneumoniae* infections, 7 European countries and Israel, epidemic periods 2011/12, 2014/15 and 2015/16

When examining the epidemic periods of 2011/12, 2014/15 and 2015/16, a clear association between the country latitude and beginning of the national epidemic period was observed (Figure 4). This was statistically significant for the 2011/12 period (p < 0.005; r^2^ = 0.92) and the 2014/15 period (p < 0.005; r^2^ = 0.84). However, significance was not achieved during the 2015/16 period (p = 0.1; r^2^ = 0.38). The association was most apparent during the major epidemic period of 2011/12 when the epidemic period was first noted in Norway (60.4^o^N) during epidemic week 22 (calendar week 40 of 2011) and was then observed to start in Israel (31^o^N) during epidemic week 43 (calendar week 9 of 2012). There was a lack of an association during the non-epidemic periods of 2012/13 and 2013/14 (data not shown).

Countries in which epidemic periods began prior to that predicted by the Moving Epidemic Method were removed from the analysis. A statistically significant association between the week in which the country epidemic began and epidemic week number was seen in 2011/12 (p < 0.005; r^2^ = 0.92) and 2014/15 (p < 0.005; r^2^ = 0.84). No significant association was seen in 2015/16 (p = 0.1; r^2^ = 0.38).

## Discussion

Our study involved assembling the largest dataset thus far of *M. pneumoniae-*positive samples and associated data on the methods used to detect *M. pneumoniae* per country. NAATs were the most common method used among the 12 countries. A variety of commercial and in-house methodologies were used in the detection of *M. pneumoniae* in our study. However, a recent study of 13 assays used across Europe, Israel and the US demonstrated comparable levels of detection of 20 *M. pneumoniae* genomes per reaction [14]. Serological methods were also commonly used to detect *M. pneumoniae* infections. The presence of an IgM response may indicate recent acquisition of infection, but it may be an unreliable marker because of documented long-term persistence of antibodies [15]. Culture-dependent methods were used by three countries. Culture has the benefit of confirming the presence of viable *M. pneumoniae* and may permit phenotypic antimicrobial susceptibility testing. However, because of the considerable time required for growth, up to 4 weeks, it does not provide results within a clinically relevant time period.

Currently, no single method is recommended for routine detection of *M. pneumoniae,* and international guidelines or control materials do not exist. Real-time PCR and serology have previously shown agreement in their ability to detect *M. pneumoniae* among samples (90% agreement), however, 7% of patients were PCR positive, with limited evidence of seroconversion [16]. No single test can reliably detect all infections. Therefore, a combined approach utilising both NAAT and serology may help to identify any potential false-negative results, with NAAT used for acute phase detection [17].

In addition to method of detection, we sought to identify if surveillance for macrolide resistance was routinely undertaken. Routine macrolide resistance monitoring was not systematically in place. This may contribute to the under-detection of resistance or reflect low levels of macrolide resistance reported across Europe [18-20]. High levels of resistance have been noted, for example, in Israel (30%) [21] and China wherein reported macrolide resistance levels are 90 to 100% [22,23]. High incidence of macrolide resistance necessitates co-ordinated surveillance across Europe. It would be of benefit to increase physicians’ knowledge of macrolide resistance rates and acquisition, as well as an understanding of the patient’s recent travel history when considering therapy. In the authors’ opinion, co-ordinated international surveillance for macrolide resistance should be considered as macrolides are the current treatment of choice in Europe.

Regardless of the methodology used to detect *M. pneumoniae*, clear seasonal trends were apparent between January 2011 and April 2016, with peaks in infection occurring between the fourth quarter of a year and first quarter of the next. To calculate the epidemic period for each season, the MEM, as described by Vega et al, was used [12]. Three clear epidemics were noted in 2011/12, 2014/15 and 2015/16. *M. pneumoniae* epidemics have been suggested to occur every 4 to 7 years, lasting for longer than one annual season in some cases [8,24]. However, in this study, based on a very large data set, the interval between epidemic occurrence was found to be 3 years from 2011/12 to 2014/15 and 1 year from 2014/15 to 2015/16. The latter epidemic period may reflect a secondary peak of cases within a 2-year epidemic span as previous epidemics have been shown to persist for some time [5,24]. Confirmation of circulating genotypes of *M. pneumoniae* from large geographical areas would be of interest to determine the microbiological nature of strains within these and epidemic periods.

For the major epidemic periods, we sought to determine any changes between the pre-epidemic, epidemic and post-epidemic periods in the reported number of detections in countries reporting both positive and negative data. The greatest rise was seen in Ireland where 18% of samples were positive for *M. pneumoniae* in the pre-epidemic period, while 30% were positive during the epidemic period in 2011/12. In Denmark, France, Slovenia and Sweden, the prevalence in the post-epidemic period was lower than that of the pre-epidemic period in 2011/12. This lower prevalence in the post-epidemic period may reflect over-sampling as a bias resulting from higher prevalence during the epidemic. This may also reflect an increase in the population burden of infection prior to an epidemic. Additional analysis is required to understand if this is the case and if monitoring of levels can be used to predict imminent epidemics.

A notable observation was the pattern of positive detections stratified by age, with detections in all age groups. *M. pneumoniae* cases are classically seen among children 5 to 14 years of age, with those under 5 years experiencing milder disease [17]. This trend was seen in Germany, Greece, Ireland and Slovenia which skew towards the younger age groups. For Greece, this can be attributed to the acquisition of samples from a tertiary children’s hospital in Athens. It should be noted that the skew towards younger age groups seen in Ireland may reflect the nature of only investigating patients up to 25 years of age. An observed peak in infection among the under 14 years age groups was followed by a second peak in the 25 to 44 years age group giving a bimodal distribution in five countries. Finally, a skew to the older age groups was seen in Hungary and Sweden. This observation is not likely to be an artefact of testing methodology whereby older people may be more likely to have existing IgM levels as both countries do not solely rely on serology. The reason for this increased detection in people above 44 years of age in two countries is unknown; it may simply reflect differences in local testing guidelines and routine practice for respiratory screening, or reflect age-based screening practices (e.g. the majority of Hungarian and Swedish samples derived from a population > 25 years of age).

The final analysis examined the association between the start of epidemic periods as calculated by the MEM model and the geographical location of the country as determined by latitude. Geographical location has not been thought to be of importance in the progression of *M. pneumoniae* infection, but our data suggests that more northern countries experience the start of epidemic periods earlier than those in the south. This association was most clear during the 2011/12 epidemic period, but also held true for the subsequent epidemic periods of 2014/15 and 2015/16. Previous national-based studies have shown epidemics to be polyclonal in nature [25-30]. Establishing whether the microbiological nature of the epidemics across Europe are clonal or not, may be beneficial and influence future sentinel surveillance design. The impact of climatic factors on *M. pneumoniae* infections has yet to be investigated.

### Limitations

A number of limitations are apparent. First, because of the variable reporting methods of each country, specific case definitions were not considered and de-duplication methodologies were not imposed but rather set at submitter-specific level. Overall, reported testing activity or testing-incidence was very different between countries (regions), and conclusions based on analysis across countries must be considered with caution. Ireland did not provide a complete dataset because they only investigated *M. pneumoniae* in patients who were 24 years of age and under. The true number of positive individuals from this population is therefore likely to be underestimated. Second, the data from some countries may not be fully representative of the country as a whole. For example, data from Germany and France was obtained from a single region within each country and how representative this is of national coverage is unknown. Third, the data examining the distribution of detections stratified by age group should be interpreted with a level of caution. The age categories did not contain equal weighting of age groups; for example, there were fewer ages encompassed in the 0 to 4 years group compared with older age groups such as the 25 to 44 years group. Finally, this data did not take the subtypes of *M. pneumoniae* that have been described into account.

### Possible clinical impact

The comparative nature of this study has highlighted a number of interesting points with regards to trends in the testing and epidemiology of *M. pneumoniae* infections. First, cases may be under-detected in countries because of limitations in the age groups examined for the infection. A number of countries showed a skew towards patients > 25 years of age and Belgium, Denmark, France, the United Kingdom (excluding Northern Ireland) and Israel showed a bimodal distribution, suggesting investigations for *M. pneumoniae,* although of importance in young children, should not be restrictive and that consistent testing guidelines are required. It would be beneficial to have an agreed international case definition for infection with *M. pneumoniae.*

This study also highlights a lack of antimicrobial resistance testing and surveillance of *M. pneumoniae,* resulting in a limited evidence base on resistance to guide therapy. Without active coordinated monitoring, it will not be possible to track changes in resistance profiles and the emergence of high-level macrolide resistant clones. There is an absence of a structured European level surveillance and resistance monitoring for this infection, despite the high levels of resistance in some global areas. In the authors’ opinion, structured surveillance should be implemented in Europe.

Finally, the observation relating to association between northern latitudes and earlier epidemic start week may suggest the need for another more focused study. It would be interesting to assess the potential value of a rapid, real-time reporting system of *M. pneumoniae* infections across Europe. Such a system could possibly aid in future epidemiological studies and resistance monitoring, and help to predict the *M. pneumoniae* epidemic season throughout the continent.

### Conclusion

As this large study demonstrates, there is currently no standardised method for detecting *M. pneumoniae* infection among patients and macrolide resistance screening is sporadic in European countries despite high levels in some areas globally. A wave of epidemics from more northern latitudes to the more southern ones occurs during epidemic years and the reason for this is not known. There is a need for testing guidelines and standardised international control material for use in testing. The potential value of a co-ordinated international surveillance and macrolide resistance monitoring system needs to be further addressed.

## ESCMID Study Group for Mycoplasma and Chlamydia Infections (ESGMAC) *Mycoplasma pneumoniae* subgroup members not listed as an individual author

**Belgium**: Nathalie Bossuyt (Service Epidemiology of Infectious Diseases, Sciensano, Brussels); Katrien Lagrou (National Reference Centre for Respiratory Pathogens, University Hospitals Leuven, Leuven)

**Cyprus**: George Mitis (Immunology Department, Nicosia General Hospital Nicosia; Maria Koliou (Department of Paediatrics, Archbishop Makarios III Hospital, Nicosia)

**Czech Republic**: Martina Havlickova, Jan Kyncl (National Institute of Public Health, Prague)

**Denmark**: Hanne-Dorthe Emborg, Marianne Voldstedlund (Statens Serum Institut, Copenhagen)

**Germany:** Colin Rae MacKenzie (Institute of Medical Microbiology and Hospital Hygiene, Heinrich-Heine-University, Duesseldorf)

**Greece**: Helena C. Maltezou, Theano Georgakopoulou (National Public Health Organization, Athens); Evangelina Petridou, Kirkira Banou (Aghia Sophia Children Hospital, Athens)

**Hungary**: Eszter Balla (Department of Reference Laboratories, National Public Health Center, Budapest)

**Ireland**: Jeff Connell, Zoe Yandle, Joanne Moran (University College Dublin, National Virus Reference Laboratory, Dublin); Karen Burns (Health Protection Surveillance Centre, Dublin)

**Israel**: Ayelet Michael-Gayego, Allon E. Moses (Department of Clinical Microbiology and infectious Diseases, Hadassah-Hebrew University Medical Centre, Jerusalem)

**Malta**: Tanya Melillo Fenech (Ministry of Health, Valletta)

**Norway**: Gabriel Ånestad, Hans Blystad, Didrik F. Vestrheim (Norwegian Institute of Public Health, Oslo)

**Portugal**: Filipe Froes (Hospital Pulido Valente and General Directorate of Health Consultant for Pneumology, Lisbon)

**Slovenia**: Darja Kese (University of Ljubljana, Faculty of Medicine

Institute of Microbiology and Immunology, Ljubljana) and Maja Socan (National Institute of Public Health, Ljubljana)

**Spain**: Rosa Cano Portero (National Center for Epidemiology, Instituto de Salud Carlos III. CIBERESP), Madrid); Sara Santos Sanz and Berta Suárez Rodríguez (Coordination for Alerts and Public Health Emergencies, Directorate General of Public Health, Ministry of Health, Social Affairs and Equality, Madrid)

**Sweden**: Anders Ternhag (Karolinska University Hospital and Public Health Agency of Sweden, Stockholm); Christian Giske (Karolinska University Hospital, Stockholm); Karin Tegmark Wisell (Public Health Agency of Sweden, Stockholm)

**United Kingdom**: Diogo Pereira Marques, Arlene Reynolds, Jim McMenamin (Health Protection Scotland, Glasgow)

## Supplementary Data

135000 Supplementary Material
