# Supplementary material for: Mycoplasma pneumoniae infections, 11 countries in Europe and Israel, 2011 to 2016
Source: Euro Surveill. 2020 Jan 16;25(2):1900112. doi: 10.2807/1560-7917.ES.2020.25.2.1900112 (PMC6976882; doi:10.2807/1560-7917.ES.2020.25.2.1900112)
Supplement: Supplementary Material [file 1900112_Supplement.pdf]

Supplementary material

S1 – Exclusion and de-duplication criteria by country. This supplementary material is hosted by *Eurosurveillance* as supporting information alongside the article *'Mycoplasma pneumoniae* infections across Europe and Israel (2011 – 2016)' on behalf of the authors who remain responsible for the accuracy and appropriateness of the content. The same standards for ethics, copyright, attributions and permissions as for the article apply. Supplements are not edited by Eurosurveillance and the journal is not responsible for the maintenance of any links or email addresses provided therein.

S1 – Exclusion and de-duplication criteria by country.

Country	De-duplication	Exclusion
Belgium	Duplicate positive tests from 1 patient in a 90-day time window were grouped and appear in the dataset as 1 positive test.	The data of the sentinel network of microbiological laboratories include "Mycoplasma-like illness" syndromes cases confirmed by one of the following criteria:<br><br>Detection of nucleic acid in a nasopharyngeal or oropharyngeal swab or in other deep respiratory specimens<br><br>Detection of seroconversion of IgG or significant increase in acute and convalescent serum sample.<br><br>Detection of *M. pneumoniae*-specific IgM antibodies.
Cyprus	Data not provided	Data not provided
Denmark	At individual level only the first mycoplasma positive test result within a season is included. If several negative mycoplasma tests are available per individual, only one negative mycoplasma test is included/counted per week. The first time an individual test positive for mycoplasma all subsequent negative and positive mycoplasma tests are excluded	At individual level only the first mycoplasma positive test result within a season is included. If several negative mycoplasma tests are available per individual, only one negative mycoplasma test is included/counted per week. The first time an individual test positive for mycoplasma all subsequent negative and positive mycoplasma tests are excluded
France	Duplicate samples from the same patient (eg. with NAAT and serology) was included as a single category : Included as serology	None given

Germany	None given	None given
Greece	None given	None given
Hungary	None given	None given
Ireland	None given	None given
Israel	Only NAAT	None
Malta	Data not provided	Data not provided
Netherlands	Not applicable	Sentinel surveillance in place, methodology and age absent, data not provided
Norway	None given	None given
Poland	Data not provided	Data not provided
Slovakia	Data not provided	Data not provided
Slovenia	Duplicate samples from the same patient was included as a single case	Serology excluded
Spain	Data not provided	Data not provided
Sweden	Duplicate samples from the same patient has not been excluded.	Four of the laboratories could only deliver data from 2012 (three labs) and 2013 (one lab) and onward.
United Kingdom (excluding Northern Ireland)	Duplicate data from same patient only included if sample date >1 year between serology samples or >3 months between NAAT samples	Serology test results from samples other than blood, serum or plasma were excluded. Includes NAAT methods (coded on surveillance database as genomic/PCR/LCR detection) on respiratory samples or blood, serum or plasma only. All other methods and specimen types (including unknown) excluded.
